# Predictive modeling of perioperative patient deterioration: combining unanticipated ICU admissions and mortality for improved risk prediction

**DOI:** 10.1186/s13741-024-00420-9

**Published:** 2024-07-03

**Authors:** Tom H.G.F. Bakkes, Eveline H.J. Mestrom, Nassim Ourahou, Uzay Kaymak, Paulo J. de Andrade Serra, Massimo Mischi, Arthur R. Bouwman, Simona Turco

**Affiliations:** 1 Electrical Engineering, Eindhoven University of Technology, Eindhoven, The Netherlands; 2 Anesthesiology, Catharina Ziekenhuis Eindhoven, Eindhoven, The Netherlands; 3 Mathematics, Vrije Universiteit Amsterdam, Amsterdam, The Netherlands

**Keywords:** Perioperative patient deterioration, Unanticipated ICU admission, In-hospital mortality, Predictive modeling, Univariate analysis, Multivariate analysis

## Abstract

**Objective:**

This paper presents a comprehensive analysis of perioperative patient deterioration by developing predictive models that evaluate unanticipated ICU admissions and in-hospital mortality both as distinct and combined outcomes.

**Materials and Methods:**

With less than 1% of cases resulting in at least one of these outcomes, we investigated 98 features to identify their role in predicting patient deterioration, using univariate analyses. Additionally, multivariate analyses were performed by employing logistic regression (LR) with LASSO regularization. We also assessed classification models, including non-linear classifiers like Support Vector Machines, Random Forest, and XGBoost.

**Results:**

During evaluation, careful attention was paid to the data imbalance therefore multiple evaluation metrics were used, which are less sensitive to imbalance. These metrics included the area under the receiver operating characteristics, precision-recall and kappa curves, and the precision, sensitivity, kappa, and F1-score. Combining unanticipated ICU admissions and mortality into a single outcome improved predictive performance overall. However, this led to reduced accuracy in predicting individual forms of deterioration, with LR showing the best performance for the combined prediction.

**Discussion:**

The study underscores the significance of specific perioperative features in predicting patient deterioration, especially revealed by univariate analysis. Importantly, interpretable models like logistic regression outperformed complex classifiers, suggesting their practicality. Especially, when combined in an ensemble model for predicting multiple forms of deterioration. These findings were mostly limited by the large imbalance in data as post-operative deterioration is a rare occurrence. Future research should therefore focus on capturing more deterioration events and possibly extending validation to multi-center studies.

**Conclusions:**

This work demonstrates the potential for accurate prediction of perioperative patient deterioration, highlighting the importance of several perioperative features and the practicality of interpretable models like logistic regression, and ensemble models for the prediction of several outcome types. In future clinical practice these data-driven prediction models might form the basis for post-operative risk stratification by providing an evidence-based assessment of risk.

**Supplementary Information:**

The online version contains supplementary material available at 10.1186/s13741-024-00420-9.

## Introduction

Delayed detection of severe deterioration is a substantial risk for postoperative patients and may lead to unanticipated admissions to the intensive care unit (ICU) (Frost et al. 2009; Romero-Brufau et al. 2021). In current clinical practice, postoperative patients are transferred to either the ward or ICU after surgery, and the post-operative destination is often determined before surgery (Oakland et al. 2021). Generally, patients who are considered low-risk are transferred to the ward. However, around 1% of these patients will experience an unanticipated ICU admission (Petersen Tym et al. 2017). Notably, unanticipated ICU admissions have been associated with a higher mortality rate than in patients with planned ICU admissions and patients with no ICU admissions (Pearse et al. 2012). Therefore, timely allocation of high-risk patients to high-acuity facilities such as the ICU could provide a positive impact on the recovery process of the patient (Mapp et al. 2013; Akkermans et al. 2020). These decisions can be supported by tools capable of risk-stratification during the planning of perioperative care (Ludbrook and Goldsman 2017; Petersen Tym et al. 2017; Varghese et al. 2018; Grigorescu et al. 2021; Adeleke et al. 2021).

Previous research on data-driven prediction of postoperative mortality has demonstrated improved performance when using intraoperative features (Yan et al. 2022; Mestrom et al. 2023; Biccard and Rodseth 2013). These are features that are measured during surgery such as vital signs, medication, and laboratory values. Similarly, studies investigating the prediction of unanticipated ICU admissions have yielded comparable findings. Initial research indicated that higher age and advanced American Society of Anesthesiology (ASA) scores were associated with a greater likelihood of experiencing unanticipated ICU admissions (Quinn et al. 2017). Subsequent investigations highlighted multiple relevant factors during surgery and in the postanesthesia care unit (PACU) (Petersen Tym et al. 2017). In addition to age and ASA scores, these studies revealed that the duration of surgery, PACU stay, and comorbidities also played predictive roles in unanticipated ICU admissions. Leveraging these findings, a multivariate logistic regression model was constructed, achieving an impressive area under the receiver operating characteristic curve (AUROC) of 90% for predicting unanticipated ICU admissions (Petersen Tym et al. 2017). However, it is important to note that the study population in that research was relatively small and unbalanced, comprising only 747 patients, of which only 7 patients experienced an unanticipated ICU admission.

Mestrom et al. expanded upon this research by creating a more extensive dataset consisting of 25,296 patients (Mestrom et al. 2023). Utilizing this dataset, they constructed prediction models based on logistic regression, similar to previous studies. These models were trained to predict postoperative deterioration, specifically defined as an unanticipated ICU admission. While the models showed good performance, it was unclear how these should be translated into clinical practice.

In this study, we build on our previous work and determine the effect of mortality on the prediction of deterioration, comparing it to the effect of unplanned ICU admissions using a univariate analysis. Moreover, we examine the relative importance of different features in making these predictions and assess how the choice of features influences the prediction results. Furthermore, we developed multivariate models, encompassing both linear and non-linear approaches, to discern the potential role of non-linearity in predicting deterioration. By comparing the performance and significance of features between the models, we aim to provide actionable insights for clinicians in effectively allocating care for postoperative patients.

## Material and Methods

### Data acquisition and study population

For this study, we retrospectively collected data from Catharina Ziekenhuis in Eindhoven, the Netherlands. Ethical approval for the study was obtained from the Medical Research Ethics Committees United (MEC-U) under reference number W18.071 on 25-5-2018 The data used in this study is based on the same patient population collected by Mestrom et al. (2023). It consists of surgery with similar postoperative care protocol, therefore including cardiothoracic surgery, obstetric surgery, catheterization lab procedures, electroconvulsive therapy, and daycare procedures were excluded. The aim of this study is to identify how perioperative data can capture patients who are missed by the current standard of care. Thus, we defined our cases as unanticipated ICU admissions, indicating that patients were initially transferred to the ward before being subsequently transferred to the ICU. More detailed information on the exact exclusion criteria and definition of unanticipated ICU admissions can be found in the Methods section of Mestrom et al. (2023).

Mortality was included as an additional end-point as the death of a patient indicates a significant deterioration as well. In total, the dataset contained 25,479 admissions of which 186 had an unanticipated ICU admission and 66 had an in-hospital mortality. The overlap between these two groups was 23. Unanticipated ICU admissions and mortality were used in the analysis both separately and combined. When combined the end-point was considered true in the case a patient experiences either, an unanticipated ICU admission, in-hospital mortality, or both.

A total of 98 features were extracted throughout the different phases of the perioperative process, including 18 from preoperative screening, 37 during surgery, 24 from the PACU, and 19 encompassing the overall perioperative process. Appendix A provides a comprehensive list of these features. The selection of features was based on previous research findings and input from local clinicians regarding indicators that might demonstrate early signs of deterioration. It was crucial to ensure that these features were widely available and not dependent on specific measurements, as this would limit their applicability to certain patient populations or result in a significant amount of missing data. While efforts were made to minimize missing data, it is almost inevitable to have some missing values. In the section Preprocessing, we elaborate on our approach to handling missing data.

### Univariate Analysis

A univariate analysis was applied to determine which features were statistically significant for the prediction of patient deterioration. The univariate analysis was performed in Python by logistic regression (LR) (Christensen 1997). The *p*-value was based on the t statistic for the significance of the corresponding feature. However, because of multiple hypothesis testing, we applied the Benjamini and Hochberg procedure to minimize false discovery (Benjamini and Hochberg 1995). After correction, the false discovery rate was controlled at 5%.

### Multivariate Analysis

The individual features were combined using a multivariate analysis that employs the same LR technique. LR leverages linear relationships between input features and the outcome. To perform feature selection and regularization, we applied the Least Absolute Shrinkage and Selection Operator (LASSO) as a penalizing function. All available features were provided to the LR, and a grid search was conducted over the cost term of the LASSO, enabling the selection of the optimal feature set.

Since unanticipated ICU admissions and mortality represent distinct forms of deterioration, it is possible that different combinations of features are required to predict each outcome. This difference in features allows us to discern potential differences in modeling deterioration when considering unanticipated ICU admissions or mortality.

Additionally, we created an ensemble model by combining the two LR models trained separately on unanticipated ICU admissions and mortality. To generate a prediction for the ensemble model, the data was input into both models and the outcome was generated by taking the maximum prediction from both models. Constructing an ensemble model in this manner enables us to compare its performance with the model trained on the combined outcome of unanticipated ICU admissions and mortality. This way, we can retain separate predictions for each form of deterioration, which can hold clinical significance.

In addition to the multivariate analysis using LR, we employed classification methods capable of exploiting non-linear relationships between input features and outcomes, potentially achieving better performance. Non-linear relationships are regularly expected in medical data especially when related to deterioration (Kipnis et al. 2016; Mestrom et al. 2023). We employed three types of classifiers that are widely known and used: Support Vector Machine (SVM), Random Forest (RF), and Extreme Gradient Boosting (XGBoost). These are known for their ability to capture non-linear relationships, and handling complex healthcare data, providing a comprehensive comparison with conventional methods.

SVM divides the feature space using a hyperplane, classifying points based on which side of the hyperplane they fall. To enable non-linear classification, a kernel can be applied to expand and nonlinearly transform the feature space (James et al. 2013). In this study, we implemented a non-linear SVM using a Gaussian kernel.

RF predicts the output by constructing an ensemble of decision trees and combining their predictions (Hastie et al. 2009). In this study, a forest size of 500 trees was used.

XGBoost is a state-of-the-art, scalable tree-boosting system widely used by data scientists (Chen and Guestrin 2016). Similar to RF, XGBoost constructs an ensemble of trees. However, XGBoost employs the gradient tree boosting algorithm to train an additive model of weak learners. In this study, we set the number of trees to 500, the learning rate to 0.3, and the $\alpha$ for L1 regularization to 1.

Each classifier also had hyperparameters that were optimized using a grid-search which is explained in the section Hyperparameter optimization. All of these classifiers were implemented using the Python Package Scikit-learn except XGBoost, which was implemented using its dedicated package.

### Preprocessing

Before training and testing the classifiers, the following preprocessing steps were performed. Categorical values were encoded using one-hot encoding. During cross-validation, missing values were imputed using the median value. The median values were calculated based on the training set and were subsequently applied to the testing set as well. Finally, the features were standardized to have a zero mean and a standard deviation of one. This standardization step was performed as a final preprocessing step before training the classifiers.

### Hyperparameter optimization

Hyperparameter optimization was performed for LR, SVM, RF, and XGBoost using a grid search approach. An overview of the parameters used for each classifier can be found in Appendix B. For LR, the cost parameter (*C*) was optimized. This parameter scales the prediction loss relative to the L1 loss, with higher values corresponding to less regularization. For SVM, the optimized parameters were the cost (*C*) and the kernel scale ($\gamma$). The cost parameter determines the extent to which the loss increases when a data point is on the wrong side of the hyperplane, while the kernel scale influences how close observations need to be before affecting each other. In the case of RF, optimization involved three parameters: the maximum depth of the trees, the minimum samples required to make a split, and the minimum samples per leaf. These parameters affect the stopping criteria for tree construction (Hastie et al. 2009; James et al. 2013). For XGBoost, optimization focused on the maximum depth, minimum child weight, and $\gamma$ parameters, all of which influence the stopping criteria for constructing weak learners (Chen and Guestrin 2016). Additionally, the learning rate was tuned.

### Evaluation

Each multivariate model and classifier underwent 5-fold cross-validation, which was repeated 10 times to ensure robustness. During each step of the cross-validation process, hyperparameter optimization was conducted. For hyperparameter optimization, the training dataset was further divided into a nested train-validation split, consisting of 75% for training and 25% for validation. This approach allows for the evaluation of model performance during optimization and has been shown to provide a more conservative estimate of model performance (Tsamardinos et al. 2015).

The following metrics were computed for each test: the area under the receiver operating characteristics curve (AUROC), the area under the precision-recall curve (AUPRC), the area under the kappa curve (AUKC) (Kaymak et al. 2012), precision, sensitivity, kappa, and F1 score. While the AUROC is commonly used for evaluating machine learning models, it is less reliable when dealing with imbalanced datasets. Therefore, F1 score, kappa, AUPRC, and AUKC were included, as these metrics are better suited for imbalanced datasets (Davis and Goadrich 2006). Precision and sensitivity were included as they provide insights into the model’s reliability in clinical practice. Precision indicates how accurately the model assigns the high-risk label to patients, while sensitivity measures its ability to correctly identify high-risk patients.

Cohen’s kappa score measures accuracy corrected for chance agreement between the model output and true labels, calculated as:

1 $$\begin{aligned} k = \frac{a - p_e}{1 - p_e}, \end{aligned}$$

where *a* represents the observed accuracy, and $p_e$ represents the chance agreement between the true labels and the model output (Landis and Koch 1977). To calculate accuracy, F1 score, and kappa, a cutoff value for the prediction score was needed. This threshold was determined on the training set by selecting the threshold that assigned 1% of patients the high-risk label. This approach deals with the class imbalance and thereby prevents overpopulation of the ICU, as it aligns with the percentage of patients who eventually end up deteriorating. Additional methods to counteract the effects of the data imbalance were attempted in the form of resampling. This included the use of synthetic minority oversampling technique to 10% and was followed by random undersampling of the majority class to 50%. However, the resampling did not provide any additional benefit over the adjustment of the threshold and was therefore excluded from the final analysis. The results of the resampling can be found in Appendix C.

For the multivariate LR, the evaluation was performed with the unanticipated ICU admissions and mortality both separately and combined as the outcome. Additionally, the model trained on the combined outcome was evaluated individually for each form of deterioration. This analysis aimed to assess whether the model trained on the combined outcome retained predictive power for each individual form of deterioration.

Finally, the classifiers, along with the LR model trained on the combined outcome and the ensemble of LR models, were evaluated on the combined outcome of unanticipated ICU admissions and mortality.

### Feature Importance

To gain a deeper understanding of the distinctions between the multivariate models for unanticipated ICU admissions and mortality, we conducted an analysis of feature importance.

Feature importance was determined using a technique called Permutation Feature Importance (PFI), which assesses a feature’s significance by randomly shuffling the values in the feature, effectively breaking any association it has with the outcome label. The performance achieved with the permuted feature is then compared to the performance without permutation, revealing the feature’s importance (Breiman 2001).

This process was repeated 5 times, and the feature’s importance was calculated as the average across all repetitions over all the folds. The number of repetitions was limited to 5 due to their computational cost. Furthermore, PFI is calculated for each of the 50 folds, which already provides a stable solution. Utilizing more repetitions did not significantly alter the PFI results.

## Results

25,479 Surgeries between January 2013 and December 2017 that matched the inclusion criteria were included in this study. An unanticipated ICU admission occurred after 186 of these surgeries, and mortality occurred in 66 cases. These groups had an overlap of 23, which means deterioration occurred in 229 cases, representing less than 1% of the total number of data points. The percentage of cases is in line with previous literature (Petersen Tym et al. 2017).

An overview of all the features and the results of the univariate analysis for the unanticipated ICU admissions, mortality, and the combination of the two can be found in Appendix A. Table 1 presents the features that exhibited a difference in significance between unanticipated ICU admissions and mortality after correction for multiple comparisons.

**Table 1:** Difference in predictive Factors for Unanticipated ICU Admissions and In-Hospital Mortality

	Unanticipated ICU Admissions		In-Hospital Mortality	
Variable	coefficient	*p*-value^a^	coefficient	*p*-value^a^
Preoperative				
BMI	-0.060 (-0.218 - 0.098)	0.505	-0.836 (-1.289 - -0.383)	**0.001**
Weight of the patient	-0.024 (-0.178 - 0.130)	0.803	-0.769 (-1.182 - -0.355)	**0.001**
Diastolic blood pressure	-0.111 (-0.264 - 0.041)	0.184	-0.607 (-0.803 - -0.411)	**0.000**
History of alcohol use	-0.006 (-0.167 - 0.156)	0.965	0.335 (0.132 - 0.537)	**0.002**
History of diabetes	0.287 (0.168 - 0.406)	**0.000**	-0.179 (-0.631 - 0.273)	0.501
History of heart failure	0.194 (0.062 - 0.327)	**0.007**	0.085 (-0.225 - 0.395)	0.645
History of hypertension	0.281 (0.137 - 0.425)	**0.000**	-0.084 (-0.459 - 0.292)	0.705
History of thrombosis	0.146 (0.027 - 0.264)	**0.022**	0.095 (-0.203 - 0.392)	0.600
Surgery				
Surgery duration	0.344 (0.267 - 0.422)	**0.000**	0.128 (-0.054 - 0.309)	0.206
Elective surgery	0.102 (-0.026 - 0.231)	0.144	0.772 (0.616 - 0.929)	**0.000**
Surgery group (General)	0.559 (0.389 - 0.728)	**0.000**	0.250 (-0.001 - 0.501)	0.069
Surgery group (Gynecology)	-0.405 (-0.658 - -0.151)	**0.003**	-7.069 (-1*10^4^ - 1*10^4^)	0.999
Surgery group (Other)	-0.186 (-0.368 - -0.004)	0.060	-0.697 (-1.334 - -0.060)	**0.045**
Surgery group (Urology)	-0.315 (-0.536 - -0.093)	**0.008**	-0.064 (-0.326 - 0.198)	0.682
Maximum systolic blood pressure	0.295 (0.171 - 0.420)	**0.000**	0.186 (-0.038 - 0.409)	0.135
Minimum heart rate	0.115 (-0.024 - 0.255)	0.133	0.450 (0.245 - 0.656)	**0.000**
Instability heart rate^b^	0.154 (0.024 - 0.284)	**0.027**	-0.027 (-0.272 - 0.218)	0.863
Maximum oxygen saturation	0.027 (-0.122 - 0.175)	0.781	-0.261 (-0.421 - -0.102)	**0.002**
Median oxygen saturation	-0.078 (-0.216 - 0.060)	0.308	-0.343 (-0.523 - -0.163)	**0.000**
Minimum oxygen saturation	-0.085 (-0.218 - 0.049)	0.249	-0.422 (-0.584 - -0.260)	**0.000**
Instability oxygen saturation^b^	0.088 (-0.045 - 0.220)	0.229	0.394 (0.230 - 0.558)	**0.000**
Narcosis type (General)	-0.244 (-0.372 - -0.116)	**0.000**	-0.023 (-0.261 - 0.215)	0.877
Use of ephedrine	0.273 (0.126 - 0.421)	**0.001**	0.175 (-0.068 - 0.418)	0.196
Recovery				
Maximum diastolic blood pressure	0.144 (0.046 - 0.242)	**0.007**	0.124 (-0.062 - 0.310)	0.233
Maximum mean blood pressure	0.167 (0.052 - 0.281)	**0.007**	0.195 (0.005 - 0.385)	0.062
Maximum systolic blood pressure	0.215 (0.081 - 0.349)	**0.003**	0.240 (0.005 - 0.475)	0.062
Maximum oxygen saturation	-0.128 (-0.267 - 0.010)	0.089	-0.326 (-0.542 - -0.110)	**0.005**
Use of erythrocytes transfusion	0.111 (0.058 - 0.165)	**0.000**	0.091 (-0.014 - 0.195)	0.120
Contact with anesthesiologist	0.208 (0.109 - 0.308)	**0.000**	0.139 (-0.048 - 0.325)	0.182
Overall				
Instability heart rate^b^	0.160 (0.032 - 0.288)	**0.021**	-0.042 (-0.289 - 0.205)	0.779
Maximum oxygen saturation	-0.049 (-0.184 - 0.087)	0.524	-0.244 (-0.409 - -0.079)	**0.006**
Median oxygen saturation	-0.122 (-0.259 - 0.015)	0.103	-0.482 (-0.660 - -0.305)	**0.000**

This table offers an examination of perioperative variables concerning two outcomes: unanticipated ICU admissions and in-hospital mortality. The variables included in the table are those that exhibited significant differences in relation to these outcomes. For a comprehensive overview of group characteristics, readers are directed to Appendix A. Organized into distinct phases of patient care-Preoperative, Surgery, and Recovery-the table provides insights into the impact of each variable across these phases. For each variable, coefficients and *p*-values of the univariate analysis are presented

*P*-values lower than 0.05 are indicated using bold text

^a^The presented *p*-values underwent correction for multiple comparisons using the Benjamini-Hochberg procedure

^b^Instability refers to the difference between the minimum and maximum value

The results of the multivariate analysis can be found in Table 2. In Table 2, the performance of multivariate models is separated based on which end-point they were evaluated. Unless indicated otherwise the models were trained on the same end-point on which they were evaluated.

**Table 2:** Multi-variate analysis of unanticipated ICU admissions and in-hospital mortality

Method	AUROC	AUPRC	AUKC	Precision	Sensitivity	Kappa	F1 score
End-point: Unanticipated ICU admissions							
LR	83.4% (2.7%)	5.1% (1.9%)	2.1% (0.3%)	8.9% (4.4%)	12.5% (6.9%)	0.10 (0.05)	10.3% (5.2%)
LR (Combined)^a^	82.1% (3.1%)	5.1% (1.8%)	2.1% (0.3%)	9.0% (3.4%)	12.5% (5.3%)	0.10 (0.04)	10.3% (4.0%)
End-point: In-hospital mortality							
LR	91.8% (2.9%)	6.4% (3.4%)	1.6% (0.4%)	7.0% (3.1%)	26.8% (11.8%)	0.11 (0.05)	11.0% (4.7%)
LR (Combined)^a^	89.5% (3.2%)	4.9% (4.3%)	1.3% (0.3%)	5.9% (2.8%)	22.4% (11.0%)	0.09 (0.04)	9.2% (4.3%)
End-point: Unanticipated ICU admissions & in-hospital mortality							
LR	83.5% (2.8%)	7.2% (2.8%)	2.6% (0.3%)	12.2% (4.2%)	13.5% (5.4%)	0.12 (0.05)	12.8% (4.6%)
LR (Ensemble)^b^	84.5% (2.8%)	7.0% (2.6%)	2.7% (0.3%)	11.4% (4.2%)	12.7% (5.8%)	0.11 (0.05)	11.9% (5.2%)
SVM	82.8% (2.9%)	5.5% (2.2%)	2.4% (0.3%)	8.4% (4.4%)	8.8% (4.7%)	0.08 (0.04)	8.4% (4.3%)
XGB	79.7% (3.9%)	5.7% (2.6%)	2.2% (0.4%)	10.9% (5.5%)	8.5% (4.6%)	0.09 (0.05)	9.4% (4.8%)
RF	81.4% (2.8%)	5.1% (1.4%)	2.3% (0.3%)	9.5% (7.0%)	4.0% (3.5%)	0.05 (0.04)	5.1% (3.7%)

Area Under the Receiver Operating Characteristic curve (*AUROC*), Area Under the Precision-Recall curve (*AUPRC*), Area Under the Kappa curve (*AUKC*)

^a^The combined LR is trained on the combined outcome of unanticipated ICU admissions and mortality

^b^The ensemble LR is an ensemble model of the LR trained on unanticipated ICU admissions and the LR trained on mortality

**Fig. 1:**
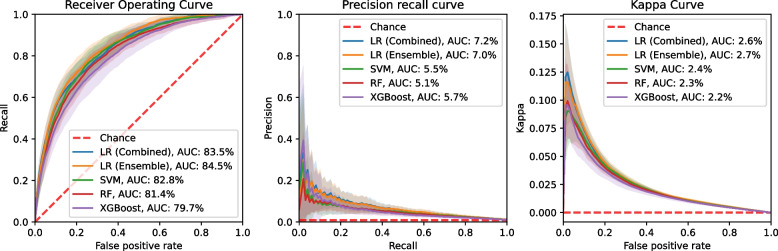
Receiver operating characteristics curve (ROC), Precision recall curve (PRC), and Kappa curve (KC) of the multivariate models and classifiers

For each of the multivariate LR models, the PFI is calculated, and Table 3 displays the feature for each multivariate model that had a mean PFI higher than its standard deviation. An additional analysis was performed using SHapley Additive exPlanations (SHAP) values, which provided similar results that can be found in Appendix D. Performance curves are displayed in Fig. 1 for all models evaluated on the combined end-point of unanticipated ICU admissions and mortality.

**Table 3:** Feature importance of multi-variate analysis

Feature	Phase	Importance
Unanticipated ICU Admissions		
ASA score	Preoperative	0.031 (0.018)
Surgery group (General)	Surgery	0.031 (0.017)
Use of phenylephrine	Surgery	0.028 (0.014)
Narcosis type (General + Epidural)	Surgery	0.019 (0.011)
Duration in PACU	Recovery	0.014 (0.014)
Minimum heart rate	Recovery	0.011 (0.009)
In-Hospital Mortality		
Elective surgery	Surgery	0.038 (0.020)
ASA score	Preoperative	0.032 (0.022)
Age	Preoperative	0.031 (0.026)
Combination		
ASA score	Preoperative	0.030 (0.016)
Surgery group (General)	Surgery	0.019 (0.012)
Age	Preoperative	0.017 (0.013)
Narcosis (General + Epidural)	Surgery	0.017 (0.010)
Use of phenylephrine	Surgery	0.016 (0.009)
Duration in PACU	Recovery	0.011 (0.009)

The importance column shows the average feature importance of all permutations over all cross-validation folds. The values between the () indicate the standard deviation

## Discussion

### Univariate Analysis

The results of the univariate analysis clearly show that each phase of the perioperative process contains valuable information for the prediction of patient deterioration since significant features were found in each phase (see Appendix A).

The univariate analysis was performed for the unanticipated ICU admissions and mortality separately and in Table 1 the features for which a difference in significance occurred between unanticipated ICU admissions and mortality can be found. From this table, it can be seen that the univariate analysis did not result in the same significance for 32 of the 98 features.

However, from the same table, we can observe that the sign of the coefficients for most of these features is the same for the unanticipated ICU admissions and mortality. Therefore, whether the feature is protective or increases the risk is independent of which form of deterioration is chosen. There are a couple of exceptions which are a history of alcohol use, diabetes and hypertension, instability of the heart during surgery, maximum saturation during surgery, and the overall instability of the heart rate. For a history of alcohol use, hypertension, instability of the heart during surgery, maximum saturation during surgery, and the overall instability of the heart rate we can see that this is because the coefficient is relatively small. Thus the sign of the coefficient could be reversed simply by chance. For the history of diabetes on the other hand this is not the case, and there it seems the risk of unanticipated ICU admissions increases while the risk of mortality decreases. It could be that diabetes increases the risk of unanticipated ICU admissions because it causes instability in blood sugar which might warrant an admission to the ICU. Fortunately, clinicians are aware of the risks associated with diabetes and proactively treat those. Thus, they might be less likely to result in in-hospital mortality.

### Multivariate Analysis

A multivariate analysis was performed using LR with LASSO regularization. Despite the presence of possible correlated features, the LR with LASSO performed well.

Table 2 highlights the importance of using metrics that are robust against imbalance. Since, if we were to only look at the AUROC it would seem that mortality is easier to predict compared to unanticipated ICU admissions, and that combining the two drives down the performance. However, looking at the AUPRC, and especially the AUKC, it shows that mortality is more difficult to predict and that combining the two outcomes results in a better performance. This suggests that combining the outcomes allows the model to leverage similarities between the two forms of deterioration effectively.

Nevertheless, combining the outcomes did result in worse performance for the prediction of the individual forms of deterioration as can be seen in Table 2. In particular, mortality is more difficult to predict for the model trained on the combined outcome. This could be due to the fact that mortality occurs less in comparison to unanticipated ICU admissions. Therefore, the model trained on the combined outcome would be biased toward the prediction of unanticipated ICU admissions. The PFI seems to confirm this as the most important feature of the mortality model does not show up for the combined model, as seen in Table 3.

As a final step in this study models were created for the prediction of deterioration. Deterioration was defined as a combination of in-hospital mortality and unanticipated ICU admissions.

The LR was already evaluated during the multivariate analysis. An ensemble LR model trained on mortality and unanticipated ICU admissions was evaluated as well since the multivariate analysis showed that the LR model trained on the combination of both loses some predictive power for the individual forms of the deterioration. In Table 2 and Fig. 1, it can be seen that LR trained on the combined outcome and the ensemble model perform similarly. However, the ensemble model has the advantage of providing separate predictions for mortality and unanticipated ICU admissions which might be clinically relevant.

The SVM, RF, and XGBoost were included since these are versatile methods capable of leveraging non-linearity to create a prediction. However, none of these methods were able to outperform the LR, as shown in Table 2 and Fig. 1.

These results show that machine learning techniques did not have an advantage in risk stratification for this dataset. One possible explanation could be a lack of non-linear relationships between the input variables and the outcome. The SVM, RF, and XGBoost would be able to leverage these relationships to improve classification. However, quantifying these non-linear relationships is not straightforward making it difficult to determine the exact cause of the lower performance. This could partly be ascribed to the possible lack of non-linear relationships in the data between the input variables and the outcome. The lack of these relationships would limit the ability of the SVM, RF, and XGBoost to leverage possible non-linear classification. Additionally, the higher complexity of these methods could lead to overfitting, which would reduce performance as well. This could be in part due to the limited size of the cases. More complex methods such as SVM, RF, and XGBoost are more difficult to fit on smaller datasets, as their flexibility requires large datasets to obtain stable results. Conventional methods such as the LR can perform nonlinear classification as well. However, this requires the application of ad-hoc non-linear transformations to the variables. This is problematic since it might not be known beforehand which variables need to be transformed and which transformation is more appropriate. The same goes for the nonlinear interaction between variables. When dealing with very data-rich processes such as the OR and PACU, the modeling becomes even more complex, since the number of variables and therefore the number of possible nonlinear interactions increases.

From Table 2 and Fig. 1, it becomes clear that all the classifiers score only slightly higher than a random classifier aware of class prevalence. Only observing the ROC might give the impression that the classifiers outperform the random classifier by a wide margin. Although the models show predictive power their clinical application might be limited as in their current form they are unable to obtain a satisfactory combination of Precision and Sensitivity as shown in Table 2 and Fig. 1. With its current performance, the models might assign the high-risk label to too many patients possibly overwhelming hospital resources. Nevertheless, the model could still be useful in highlighting high-risk patients and making health professionals aware of them.

The moderate predictive power of the multivariate models could be related to patients in the controls who were deteriorating, but proper actions taken in the ward prevented them from deteriorating further. To investigate this further, more information from the ward is needed. Possible improvements could also be gained from redefining the definition of high-risk patients since an unanticipated ICU admission already indicates a severe deterioration. A scale for the severity of the risk could be used instead. This could be achieved by defining different categories of deterioration or using the maximum early warning score obtained by the patient while on the ward.

Although the predictive power of the proposed models was only moderate, this study provided important insights into the influence of the choice of endpoint for deterioration (unanticipated ICU admission and/or mortality) and how focussing on single metrics can be deceiving. Unfortunately, many studies in risk prediction often only report the AUROC or accuracy. In Petersen Tym et al. (2017), an AUROC of 90% was reported for a linear prediction model. The imbalance in the dataset was as high as in the present study (less than 1%), with 12 unanticipated ICU admissions over 747 total observations. However, no cross-validation was performed, only a linear model was applied, backward stepping was used as a feature selection tool, and no additional metrics besides the AUROC were calculated. All these factors could lead to biased results.

Current clinical practice relies on preoperative assessment to determine the post-operative destination of the patient. One such example is the surgical outcome risk tool (SORT) (Oakland et al. 2021) This risk-stratification tool uses preoperative data to assess the risk of death within the next 30 days. The score uses ASA, urgency, high-risk specialty, the severity of surgery, cancer, and age. This study did not include high-risk specialties, the severity of surgery, and cancer, but did include the surgery group. Which was often indicated as an important feature by the PFI (see Table 3). The same agreement was found for the ASA score and age. However, this study also included surgical and post-operative features, which in some cases were marked as important by the PFI analysis as well. This is in agreement with other previous studies as these also found that perioperative features provide important information for risk stratification (Akkermans et al. 2020; Biccard and Rodseth 2013; Adeleke et al. 2021).

### Limitations

There are some limitations to this study. First, manual screening was required to label the unanticipated ICU admissions, which might make this method unsuitable for application in other hospitals. Secondly, because of the limited time available to complete the documentation of preoperative features for emergency surgery, these patients had more missing data. Third, unanticipated ICU admission is a rare event, as it occurred in only 1% of patients. This resulted in an unbalanced dataset and a limited number of cases. Further endeavors should focus on collecting more perioperative data with a larger number of cases, which would further expand the effect size of the analysis. In turn, a larger dataset might improve the fitting of the more complex methods such as SVM, RF, and XGBoost, which could improve the risk-stratification performance even further. However, this will always be the case for this type of data set, given the rarity of severe deterioration. Fourth, no external validation dataset was utilized. Local procedures and protocols might differ between hospitals, which makes the generalization of the model more difficult. Nevertheless, the univariate analysis and the variable importance highlighted some of the same features found in previous research. Fifth, data were extracted during the perioperative process, while some unanticipated ICU admissions did not occur until days later. These deteriorations might have resulted from processes after the surgery and thus might not have been correlated to the extracted data.

In addition to the data, there were a few limitations to the selected classifiers. Only a limited number of classifiers were explored. More complex classifiers such as multi-layer neural networks exist. However, given the size of the dataset, and especially the low number of unanticipated ICU admissions, these types of methods are unlikely to achieve better performance.

## Conclusion

This study proposes several models for the prediction of postoperative deterioration, defined as unanticipated ICU admission and/or mortality.

Multivariate analysis, specifically using logistic regression with LASSO regularization, demonstrated that combining unanticipated ICU admissions and mortality as a single outcome improved overall performance. However, this combination led to reduced accuracy in predicting the individual forms of deterioration. Additionally, multivariate and univariate analysis showed the importance of utilizing features from different phases of the perioperative process.

Classification models revealed that logistic regression and the ensemble model performed similarly, with the latter providing separate predictions for each outcome. More complex classifiers like SVM, RF, and XGBoost did not outperform simpler models.

The results of this study offer valuable insights for predicting patient deterioration in the perioperative setting, emphasizing the significance of specific features and the role of combining outcomes for enhanced predictive accuracy.

### Supplementary Information

**Additional file 1.** Supplementary Material 1.

## Data Availability

The datasets generated and analyzed during this study are not publicly available but are available from the corresponding author upon reasonable request.
