# Supplementary material for: Predictive modeling of perioperative patient deterioration: combining unanticipated ICU admissions and mortality for improved risk prediction
Source: Perioper Med (Lond). 2024 Jul 3;13:66. doi: 10.1186/s13741-024-00420-9 (PMC11220961; doi:10.1186/s13741-024-00420-9)
Supplement: Supplementary file 1 — Additional file 1. Supplementary Material 1. [file 13741_2024_420_MOESM1_ESM.pdf]

A Summary statistic

Table 5: Summary statistic for unanticipated ICU admission

Feature	controls	cases	coefficient	p-value	p-value*
Total number of patients	25293	186			
Preoperative					
**Age**	**55.0 (42.0 - 69.0)**	**65.0 (55.0 - 73.8)**	**0.512 (0.355 - 0.668)**	**0.000**	**0.000**
**ASA score**	**2.0 (2.0 - 2.0)**	**2.0 (2.0 - 3.0)**	**0.645 (0.496 - 0.794)**	**0.000**	**0.000**
BMI	27.1 (23.9 - 33.1)	26.7 (23.4 - 33.3)	-0.060 (-0.218 - 0.098)	0.454	0.505
Length of patient	170.0 (164.0 - 178.0)	170.0 (165.0 - 180.0)	0.056 (-0.096 - 0.208)	0.471	0.518
Weight of the patient	80.0 (69.0 - 98.0)	80.0 (66.2 - 98.0)	-0.024 (-0.178 - 0.130)	0.758	0.803
**Sex**	**10001 (39.5%)**	**97 (52.2%)**	**0.250 (0.109 - 0.391)**	**0.001**	**0.001**
Diastolic blood pressure	80.0 (70.0 - 85.0)	78.0 (70.0 - 85.5)	-0.111 (-0.264 - 0.041)	0.152	0.184
Systolic blood pressure	130.0 (120.0 - 145.0)	135.0 (122.0 - 145.0)	0.024 (-0.130 - 0.177)	0.763	0.803
History of alcohol use	1197 (5.4%)	8 (5.3%)	-0.006 (-0.167 - 0.156)	0.946	0.965
History of cerebrovascular accident	888 (4.0%)	10 (6.6%)	0.103 (-0.023 - 0.230)	0.110	0.137
History of COPD	2749 (12.4%)	20 (13.2%)	0.023 (-0.132 - 0.179)	0.770	0.803
**History of diabetes**	**2465 (11.1%)**	**36 (23.7%)**	**0.287 (0.168 - 0.406)**	**0.000**	**0.000**
**History of heart failure**	**3201 (14.4%)**	**35 (22.6%)**	**0.194 (0.062 - 0.327)**	**0.004**	**0.007**
History of heart rhythm problems	2834 (12.8%)	25 (16.4%)	0.099 (-0.045 - 0.243)	0.176	0.210
**History of hypertension**	**5948 (26.8%)**	**62 (40.8%)**	**0.281 (0.137 - 0.425)**	**0.000**	**0.000**
History of kidney failure	847 (3.8%)	8 (5.3%)	0.065 (-0.072 - 0.202)	0.355	0.405
History of smoking	4786 (21.6%)	37 (24.3%)	0.063 (-0.090 - 0.217)	0.417	0.469
**History of thrombosis**	**987 (4.5%)**	**13 (8.6%)**	**0.146 (0.027 - 0.264)**	**0.016**	**0.022**
Surgery					
**Duration in OR**	**77.0 (54.0 - 113.0)**	**128.0 (86.0 - 175.5)**	**0.407 (0.329 - 0.484)**	**0.000**	**0.000**
**Surgery duration**	**52.0 (32.0 - 80.0)**	**90.0 (63.0 - 127.5)**	**0.344 (0.267 - 0.422)**	**0.000**	**0.000**
Elective surgery	2878 (11.4%)	28 (15.1%)	0.102 (-0.026 - 0.231)	0.118	0.144
**Surgery group (General)**	**12984 (51.3%)**	**142 (76.3%)**	**0.559 (0.389 - 0.728)**	**0.000**	**0.000**
**Surgery group (Gynecology)**	**2759 (10.9%)**	**6 (3.2%)**	**-0.405 (-0.658 - -0.151)**	**0.002**	**0.003**
**Surgery group (Orthopedic)**	**3788 (15.0%)**	**17 (9.1%)**	**-0.200 (-0.378 - -0.022)**	**0.028**	**0.038**
Surgery group (Other)	2987 (11.8%)	13 (7.0%)	-0.186 (-0.368 - -0.004)	0.045	0.060
**Surgery group (Urology)**	**2775 (11.0%)**	**8 (4.3%)**	**-0.315 (-0.536 - -0.093)**	**0.005**	**0.008**
**Arterial line**	**720 (2.8%)**	**32 (17.2%)**	**0.332 (0.266 - 0.397)**	**0.000**	**0.000**
**Maximum diastolic blood pressure**	**87.0 (78.0 - 97.0)**	**90.0 (81.0 - 104.0)**	**0.256 (0.187 - 0.326)**	**0.000**	**0.000**
**Minimum diastolic blood pressure**	**43.0 (37.0 - 50.0)**	**37.5 (31.0 - 44.0)**	**-0.606 (-0.758 - -0.453)**	**0.000**	**0.000**
**Instability diastolic blood pressure**	**43.0 (32.0 - 54.0)**	**51.0 (43.0 - 66.0)**	**0.302 (0.242 - 0.363)**	**0.000**	**0.000**
**Maximum mean blood pressure**	**114.0 (102.0 - 128.0)**	**119.0 (106.2 - 133.0)**	**0.287 (0.205 - 0.370)**	**0.000**	**0.000**
**Minimum mean blood pressure**	**61.0 (53.0 - 70.0)**	**52.0 (45.0 - 61.0)**	**-0.626 (-0.754 - -0.498)**	**0.000**	**0.000**
**Instability mean blood pressure**	**52.0 (38.0 - 68.0)**	**65.0 (52.0 - 82.8)**	**0.366 (0.298 - 0.435)**	**0.000**	**0.000**
**Maximum systolic blood pressure**	**157.0 (138.0 - 177.0)**	**164.0 (144.0 - 183.0)**	**0.295 (0.171 - 0.420)**	**0.000**	**0.000**
**Minimum systolic blood pressure**	**82.0 (71.0 - 94.0)**	**71.0 (59.2 - 82.0)**	**-0.652 (-0.785 - -0.519)**	**0.000**	**0.000**
**Instability systolic blood pressure**	**73.0 (50.0 - 97.0)**	**90.0 (73.0 - 117.8)**	**0.487 (0.387 - 0.587)**	**0.000**	**0.000**
**Maximum heart rate**	**95.0 (83.0 - 107.0)**	**101.0 (89.2 - 112.0)**	**0.210 (0.084 - 0.336)**	**0.001**	**0.002**
**Median heart rate**	**66.0 (59.0 - 75.0)**	**71.0 (62.0 - 80.0)**	**0.314 (0.186 - 0.442)**	**0.000**	**0.000**
Minimum heart rate	54.0 (48.0 - 61.0)	55.0 (49.0 - 63.8)	0.115 (-0.024 - 0.255)	0.106	0.133
**Instability heart rate**	**40.0 (29.0 - 52.0)**	**42.0 (33.0 - 53.0)**	**0.154 (0.024 - 0.284)**	**0.020**	**0.027**
Maximum oxygen saturation	99.9 (99.1 - 100.0)	99.8 (99.2 - 100.0)	0.027 (-0.122 - 0.175)	0.725	0.781
Median oxygen saturation	97.7 (96.7 - 98.4)	97.6 (96.5 - 98.4)	-0.078 (-0.216 - 0.060)	0.267	0.308
Minimum oxygen saturation	93.4 (89.4 - 95.4)	92.9 (88.7 - 95.1)	-0.085 (-0.218 - 0.049)	0.214	0.249
Instability oxygen saturation	5.9 (4.0 - 9.9)	6.6 (4.4 - 10.6)	0.088 (-0.045 - 0.220)	0.194	0.229
**Narcosis type (General)**	**19432 (76.8%)**	**121 (65.1%)**	**-0.244 (-0.372 - -0.116)**	**0.000**	**0.000**
**Narcosis type (General + Epidural)**	**1079 (4.3%)**	**54 (29.0%)**	**0.457 (0.391 - 0.523)**	**0.000**	**0.000**
Narcosis type (Other)	1056 (4.2%)	2 (1.1%)	-0.277 (-0.555 - 0.001)	0.051	0.067
**Narcosis type (Spinal)**	**3726 (14.7%)**	**9 (4.8%)**	**-0.433 (-0.670 - -0.195)**	**0.000**	**0.001**
**Use of erythrocytes transfusion**	**128 (0.5%)**	**9 (4.8%)**	**0.168 (0.118 - 0.219)**	**0.000**	**0.000**
**Use of plasma**	**14 (0.1%)**	**1 (0.5%)**	**0.055 (0.006 - 0.105)**	**0.028**	**0.038**
**Use of ephedrine**	**11951 (47.3%)**	**113 (60.8%)**	**0.273 (0.126 - 0.421)**	**0.000**	**0.001**
**Use of phenylephrine**	**4510 (17.8%)**	**98 (52.7%)**	**0.630 (0.518 - 0.741)**	**0.000**	**0.000**
**Use of vasopressor pump**	**1198 (4.7%)**	**17 (9.1%)**	**0.150 (0.043 - 0.257)**	**0.006**	**0.009**
**Use of thrombocytes**	**7 (0.0%)**	**1 (0.5%)**	**0.053 (0.015 - 0.090)**	**0.006**	**0.008**
**Infusion of hydroxyethyl starch**	**638 (2.5%)**	**21 (11.3%)**	**0.253 (0.180 - 0.326)**	**0.000**	**0.000**
Recovery					
**Duration in PACU**	**74.0 (59.0 - 96.0)**	**103.0 (77.0 - 149.0)**	**0.517 (0.438 - 0.596)**	**0.000**	**0.000**
**Arterial line**	**478 (1.9%)**	**25 (13.4%)**	**0.290 (0.230 - 0.350)**	**0.000**	**0.000**
**Maximum diastolic blood pressure**	**81.0 (72.0 - 90.0)**	**82.0 (71.0 - 94.0)**	**0.144 (0.046 - 0.242)**	**0.004**	**0.007**
**Minimum diastolic blood pressure**	**63.0 (54.0 - 72.0)**	**59.0 (48.8 - 69.0)**	**-0.328 (-0.465 - -0.191)**	**0.000**	**0.000**
**Instability blood pressure**	**16.0 (11.0 - 23.0)**	**21.0 (14.0 - 30.2)**	**0.189 (0.128 - 0.250)**	**0.000**	**0.000**
**Maximum mean blood pressure**	**107.0 (96.0 - 119.0)**	**110.0 (97.8 - 123.0)**	**0.167 (0.052 - 0.281)**	**0.004**	**0.007**
**Minimum mean blood pressure**	**87.0 (77.0 - 98.0)**	**81.0 (69.0 - 94.2)**	**-0.338 (-0.464 - -0.212)**	**0.000**	**0.000**
**Instability mean blood pressure**	**18.0 (13.0 - 27.0)**	**25.0 (17.0 - 40.2)**	**0.225 (0.164 - 0.287)**	**0.000**	**0.000**
**Maximum systolic blood pressure**	**148.0 (131.0 - 166.0)**	**154.0 (136.0 - 171.2)**	**0.215 (0.081 - 0.349)**	**0.002**	**0.003**
**Minimum systolic blood pressure**	**119.0 (105.0 - 136.0)**	**114.5 (92.0 - 131.0)**	**-0.371 (-0.503 - -0.240)**	**0.000**	**0.000**
**Instability systolic blood pressure**	**25.0 (17.0 - 37.0)**	**37.0 (24.8 - 58.0)**	**0.320 (0.252 - 0.387)**	**0.000**	**0.000**
**Maximum heart rate**	**88.0 (77.0 - 100.0)**	**95.5 (84.0 - 106.0)**	**0.322 (0.199 - 0.444)**	**0.000**	**0.000**
**Median heart rate**	**71.5 (63.0 - 81.5)**	**79.5 (71.0 - 89.0)**	**0.456 (0.325 - 0.586)**	**0.000**	**0.000**
**Minimum heart rate**	**63.0 (55.0 - 73.0)**	**70.0 (62.0 - 80.0)**	**0.458 (0.326 - 0.590)**	**0.000**	**0.000**
Instability heart rate	22.0 (15.0 - 31.0)	22.0 (15.0 - 30.0)	-0.017 (-0.167 - 0.133)	0.824	0.850
Maximum oxygen saturation	98.9 (97.8 - 99.9)	98.8 (97.6 - 99.9)	-0.128 (-0.267 - 0.010)	0.069	0.089
**Median oxygen saturation**	**96.7 (95.2 - 98.1)**	**96.5 (94.6 - 97.8)**	**-0.190 (-0.332 - -0.047)**	**0.009**	**0.013**
**Minimum oxygen saturation**	**91.3 (88.0 - 93.8)**	**89.2 (84.9 - 92.2)**	**-0.295 (-0.408 - -0.182)**	**0.000**	**0.000**
**Instability oxygen saturation**	**7.0 (4.8 - 10.5)**	**8.8 (6.4 - 13.2)**	**0.269 (0.155 - 0.383)**	**0.000**	**0.000**
**Use of erythrocytes transfusion**	**67 (0.3%)**	**4 (2.2%)**	**0.111 (0.058 - 0.165)**	**0.000**	**0.000**
Use of plasma	5 (0.0%)	0 (0.0%)	-0.233 (-359.145 - 358.679)	0.999	0.999
Use of thrombocytes	4 (0.0%)	0 (0.0%)	-0.209 (-359.128 - 358.710)	0.999	0.999
**Infusion of hydroxyethyl starch**	**109 (0.4%)**	**6 (3.2%)**	**0.137 (0.081 - 0.193)**	**0.000**	**0.000**
**Contact with anesthesiologist**	**1295 (5.1%)**	**22 (12.2%)**	**0.208 (0.109 - 0.308)**	**0.000**	**0.000**
Overall					
**Total perioperative time**	**160.0 (126.0 - 207.0)**	**241.5 (175.0 - 325.2)**	**0.550 (0.468 - 0.632)**	**0.000**	**0.000**
**Arterial line**	**740 (2.9%)**	**32 (17.2%)**	**0.331 (0.264 - 0.397)**	**0.000**	**0.000**
**Maximum diastolic blood pressure**	**90.0 (81.0 - 100.0)**	**93.5 (84.0 - 108.0)**	**0.262 (0.193 - 0.331)**	**0.000**	**0.000**
**Minimum diastolic blood pressure**	**43.0 (37.0 - 50.0)**	**37.0 (31.0 - 44.0)**	**-0.554 (-0.700 - -0.408)**	**0.000**	**0.000**
**Instability diastolic blood pressure**	**46.0 (36.0 - 58.0)**	**55.0 (44.0 - 72.0)**	**0.299 (0.237 - 0.361)**	**0.000**	**0.000**

Continue on the next page

Feature	controls	cases	coefficient	p-value	p-value*
**Maximum mean blood pressure**	**119.0 (107.0 - 131.0)**	**123.5 (112.0 - 140.0)**	**0.298 (0.218 - 0.377)**	**0.000**	**0.000**
**Minimum mean blood pressure**	**60.0 (53.0 - 69.0)**	**52.0 (44.0 - 61.0)**	**-0.575 (-0.696 - -0.454)**	**0.000**	**0.000**
**Instability mean blood pressure**	**57.0 (43.0 - 72.0)**	**71.0 (57.0 - 89.0)**	**0.359 (0.291 - 0.427)**	**0.000**	**0.000**
**Maximum systolic blood pressure**	**163.0 (145.0 - 184.0)**	**171.0 (153.2 - 198.0)**	**0.338 (0.218 - 0.458)**	**0.000**	**0.000**
**Minimum systolic blood pressure**	**81.0 (71.0 - 93.0)**	**70.0 (59.0 - 81.8)**	**-0.600 (-0.723 - -0.477)**	**0.000**	**0.000**
**Instability systolic blood pressure**	**81.0 (58.0 - 104.0)**	**101.0 (81.0 - 129.8)**	**0.484 (0.388 - 0.579)**	**0.000**	**0.000**
**Maximum heart rate**	**97.0 (86.0 - 110.0)**	**103.0 (92.2 - 114.0)**	**0.240 (0.120 - 0.361)**	**0.000**	**0.000**
**Median heart rate**	**69.0 (62.0 - 78.0)**	**75.2 (68.0 - 84.9)**	**0.402 (0.276 - 0.529)**	**0.000**	**0.000**
**Minimum heart rate**	**53.0 (47.0 - 60.0)**	**55.0 (48.2 - 63.0)**	**0.192 (0.056 - 0.328)**	**0.006**	**0.008**
**Instability heart rate**	**43.0 (32.0 - 55.0)**	**47.0 (36.0 - 57.0)**	**0.160 (0.032 - 0.288)**	**0.015**	**0.021**
Maximum oxygen saturation	100.0 (99.3 - 100.0)	100.0 (99.3 - 100.0)	-0.049 (-0.184 - 0.087)	0.482	0.524
Median oxygen saturation	97.3 (96.3 - 98.2)	97.1 (95.8 - 98.1)	-0.122 (-0.259 - 0.015)	0.081	0.103
**Minimum oxygen saturation**	**89.8 (85.6 - 92.8)**	**87.9 (84.1 - 91.6)**	**-0.204 (-0.328 - -0.080)**	**0.001**	**0.002**
**Instability oxygen saturation**	**9.7 (6.7 - 14.0)**	**11.1 (7.7 - 15.5)**	**0.198 (0.074 - 0.323)**	**0.002**	**0.003**

Table 6: Summary statistic of in-hospital mortality

Feature	controls	cases	coefficient	p-value	p-value*
Total number of patients	25413	66			
Preoperative					
**Age**	**56.0 (42.0 - 69.0)**	**78.0 (69.0 - 84.0)**	**1.539 (1.178 - 1.901)**	**0.000**	**0.000**
**ASA score**	**2.0 (2.0 - 2.0)**	**3.0 (2.0 - 3.0)**	**1.336 (1.044 - 1.627)**	**0.000**	**0.000**
**BMI**	**27.1 (23.9 - 33.1)**	**24.1 (22.5 - 26.0)**	**-0.836 (-1.289 - -0.383)**	**0.000**	**0.001**
Length of patient	170.0 (164.0 - 178.0)	170.0 (160.5 - 180.0)	-0.086 (-0.379 - 0.206)	0.562	0.619
**Weight of the patient**	**80.0 (69.0 - 98.0)**	**71.8 (60.2 - 80.8)**	**-0.769 (-1.182 - -0.355)**	**0.000**	**0.001**
**Sex**	**10059 (39.6%)**	**39 (59.1%)**	**0.387 (0.146 - 0.627)**	**0.002**	**0.003**
**Diastolic blood pressure**	**80.0 (70.0 - 85.0)**	**70.0 (60.2 - 74.0)**	**-0.607 (-0.803 - -0.411)**	**0.000**	**0.000**
Systolic blood pressure	130.0 (120.0 - 145.0)	131.0 (118.5 - 143.0)	-0.199 (-0.500 - 0.102)	0.195	0.233
**History of alcohol use**	**1199 (5.4%)**	**6 (20.0%)**	**0.335 (0.132 - 0.537)**	**0.001**	**0.002**
History of cerebrovascular accident	895 (4.0%)	3 (10.0%)	0.192 (-0.042 - 0.427)	0.108	0.140
History of COPD	2762 (12.4%)	7 (23.3%)	0.253 (-0.026 - 0.532)	0.075	0.101
History of diabetes	2499 (11.2%)	2 (6.7%)	-0.179 (-0.631 - 0.273)	0.438	0.501
History of heart failure	3230 (14.4%)	6 (17.6%)	0.085 (-0.225 - 0.395)	0.592	0.645
History of heart rhythm problems	2853 (12.8%)	6 (20.0%)	0.179 (-0.120 - 0.478)	0.242	0.285
History of hypertension	6003 (26.9%)	7 (23.3%)	-0.084 (-0.459 - 0.292)	0.662	0.705
History of kidney failure	853 (3.8%)	2 (6.7%)	0.113 (-0.163 - 0.388)	0.423	0.493
History of smoking	4813 (21.6%)	10 (33.3%)	0.245 (-0.068 - 0.558)	0.125	0.159
History of thrombosis	998 (4.5%)	2 (6.9%)	0.095 (-0.203 - 0.392)	0.533	0.600
Surgery					
**Duration in OR**	**77.0 (55.0 - 113.0)**	**98.5 (69.0 - 129.8)**	**0.216 (0.043 - 0.390)**	**0.014**	**0.023**
Surgery duration	52.0 (32.0 - 80.0)	66.5 (35.5 - 96.5)	0.128 (-0.054 - 0.309)	0.168	0.206
**Elective surgery**	**2867 (11.3%)**	**39 (59.1%)**	**0.772 (0.616 - 0.929)**	**0.000**	**0.000**
Surgery group (General)	13084 (51.5%)	42 (63.6%)	0.250 (-0.001 - 0.501)	0.051	0.069
Surgery group (Gynecology)	2765 (10.9%)	0 (0.0%)	-7.069 (-11230.271 - 11216.132)	0.999	0.999
**Surgery group (Orthopedic)**	**3788 (14.9%)**	**17 (25.8%)**	**0.244 (0.047 - 0.441)**	**0.015**	**0.024**
**Surgery group (Other)**	**2999 (11.8%)**	**1 (1.5%)**	**-0.697 (-1.334 - -0.060)**	**0.032**	**0.045**
Surgery group (Urology)	2777 (10.9%)	6 (9.1%)	-0.064 (-0.326 - 0.198)	0.633	0.682
**Arterial line**	**740 (2.9%)**	**12 (18.2%)**	**0.339 (0.232 - 0.446)**	**0.000**	**0.000**
**Maximum diastolic blood pressure**	**87.0 (78.0 - 97.0)**	**81.0 (70.0 - 97.0)**	**0.188 (0.045 - 0.330)**	**0.010**	**0.016**
**Minimum diastolic blood pressure**	**43.0 (37.0 - 50.0)**	**34.0 (28.0 - 40.0)**	**-0.947 (-1.169 - -0.726)**	**0.000**	**0.000**
**Instability diastolic blood pressure**	**43.0 (32.0 - 54.0)**	**46.0 (34.0 - 61.0)**	**0.291 (0.191 - 0.391)**	**0.000**	**0.000**
**Maximum mean blood pressure**	**115.0 (102.0 - 128.0)**	**111.0 (96.0 - 129.0)**	**0.203 (0.037 - 0.370)**	**0.017**	**0.026**
**Minimum mean blood pressure**	**61.0 (53.0 - 69.0)**	**49.0 (38.0 - 56.0)**	**-0.865 (-1.048 - -0.683)**	**0.000**	**0.000**
**Instability mean blood pressure**	**52.0 (38.0 - 68.0)**	**62.0 (44.0 - 79.0)**	**0.365 (0.256 - 0.474)**	**0.000**	**0.000**
Maximum systolic blood pressure	157.0 (138.0 - 177.0)	156.0 (135.0 - 186.0)	0.186 (-0.038 - 0.409)	0.103	0.135
**Minimum systolic blood pressure**	**81.0 (71.0 - 94.0)**	**63.0 (54.0 - 75.0)**	**-0.910 (-1.099 - -0.720)**	**0.000**	**0.000**
**Instability systolic blood pressure**	**73.0 (51.0 - 97.0)**	**87.0 (64.0 - 117.0)**	**0.527 (0.371 - 0.683)**	**0.000**	**0.000**
**Maximum heart rate**	**95.0 (83.0 - 108.0)**	**96.5 (85.0 - 111.0)**	**0.237 (0.033 - 0.442)**	**0.023**	**0.035**
**Median heart rate**	**66.0 (59.0 - 75.0)**	**75.5 (66.5 - 85.8)**	**0.603 (0.418 - 0.787)**	**0.000**	**0.000**
**Minimum heart rate**	**54.0 (48.0 - 61.0)**	**58.0 (52.0 - 69.8)**	**0.450 (0.245 - 0.656)**	**0.000**	**0.000**
Instability heart rate	40.0 (29.0 - 52.0)	36.0 (24.0 - 47.8)	-0.027 (-0.272 - 0.218)	0.827	0.863
**Maximum oxygen saturation**	**99.9 (99.1 - 100.0)**	**99.8 (99.1 - 100.0)**	**-0.261 (-0.421 - -0.102)**	**0.001**	**0.002**
**Median oxygen saturation**	**97.7 (96.7 - 98.4)**	**97.9 (96.2 - 98.8)**	**-0.343 (-0.523 - -0.163)**	**0.000**	**0.000**
**Minimum oxygen saturation**	**93.4 (89.4 - 95.4)**	**91.1 (84.4 - 93.8)**	**-0.422 (-0.584 - -0.260)**	**0.000**	**0.000**
**Instability oxygen saturation**	**5.9 (4.0 - 9.9)**	**8.2 (5.4 - 14.9)**	**0.394 (0.230 - 0.558)**	**0.000**	**0.000**
Narcosis type (General)	19503 (76.7%)	50 (75.8%)	-0.023 (-0.261 - 0.215)	0.850	0.877
**Narcosis type (General + Epidural)**	**1124 (4.4%)**	**9 (13.6%)**	**0.253 (0.108 - 0.398)**	**0.001**	**0.001**
Narcosis type (Other)	1054 (4.1%)	4 (6.1%)	0.080 (-0.122 - 0.282)	0.440	0.501
**Narcosis type (Spinal)**	**3732 (14.7%)**	**3 (4.5%)**	**-0.455 (-0.864 - -0.045)**	**0.030**	**0.043**
**Use of erythrocytes transfusion**	**130 (0.5%)**	**7 (10.6%)**	**0.230 (0.171 - 0.288)**	**0.000**	**0.000**
**Use of plasma**	**14 (0.1%)**	**1 (1.5%)**	**0.081 (0.031 - 0.130)**	**0.001**	**0.003**
Use of ephedrine	12027 (47.3%)	37 (56.1%)	0.175 (-0.068 - 0.418)	0.158	0.196
**Use of phenylephrine**	**4565 (18.0%)**	**43 (65.2%)**	**0.825 (0.630 - 1.021)**	**0.000**	**0.000**
**Use of vasopressor pump**	**1195 (4.7%)**	**20 (30.3%)**	**0.464 (0.351 - 0.576)**	**0.000**	**0.000**
**Use of thrombocytes**	**7 (0.0%)**	**1 (1.5%)**	**0.071 (0.034 - 0.109)**	**0.000**	**0.000**
**Infusion of hydroxyethyl starch**	**650 (2.6%)**	**9 (13.6%)**	**0.285 (0.173 - 0.397)**	**0.000**	**0.000**
Recovery					
**Duration in PACU**	**74.0 (59.0 - 96.0)**	**83.0 (60.0 - 112.0)**	**0.294 (0.119 - 0.470)**	**0.001**	**0.002**
**Arterial line**	**494 (1.9%)**	**9 (13.6%)**	**0.289 (0.190 - 0.387)**	**0.000**	**0.000**
Maximum diastolic blood pressure	81.0 (72.0 - 90.0)	71.0 (65.5 - 89.5)	0.124 (-0.062 - 0.310)	0.192	0.233
**Minimum diastolic blood pressure**	**63.0 (54.0 - 72.0)**	**53.0 (41.0 - 63.0)**	**-0.644 (-0.851 - -0.436)**	**0.000**	**0.000**
**Instability blood pressure**	**16.0 (11.0 - 23.0)**	**20.0 (11.0 - 29.5)**	**0.225 (0.139 - 0.311)**	**0.000**	**0.000**
Maximum mean blood pressure	107.0 (96.0 - 119.0)	106.0 (95.5 - 121.5)	0.195 (0.005 - 0.385)	0.044	0.062
**Minimum mean blood pressure**	**87.0 (77.0 - 98.0)**	**75.0 (63.5 - 86.5)**	**-0.575 (-0.757 - -0.394)**	**0.000**	**0.000**
**Instability mean blood pressure**	**18.0 (13.0 - 27.0)**	**27.0 (17.0 - 47.0)**	**0.274 (0.192 - 0.356)**	**0.000**	**0.000**
Maximum systolic blood pressure	148.0 (131.0 - 166.0)	149.0 (138.5 - 181.5)	0.240 (0.005 - 0.475)	0.045	0.062
**Minimum systolic blood pressure**	**119.0 (105.0 - 136.0)**	**105.0 (85.5 - 121.5)**	**-0.555 (-0.759 - -0.351)**	**0.000**	**0.000**
**Instability systolic blood pressure**	**25.0 (17.0 - 37.0)**	**41.0 (25.0 - 64.0)**	**0.364 (0.267 - 0.461)**	**0.000**	**0.000**
**Maximum heart rate**	**88.0 (77.0 - 100.0)**	**91.0 (80.0 - 105.0)**	**0.313 (0.102 - 0.524)**	**0.004**	**0.006**
**Median heart rate**	**72.0 (63.0 - 82.0)**	**81.0 (72.5 - 90.0)**	**0.561 (0.344 - 0.778)**	**0.000**	**0.000**
**Minimum heart rate**	**63.0 (55.0 - 73.0)**	**73.0 (62.0 - 81.0)**	**0.496 (0.267 - 0.725)**	**0.000**	**0.000**
Instability heart rate	22.0 (15.0 - 31.0)	16.0 (11.0 - 26.0)	-0.084 (-0.361 - 0.193)	0.551	0.613
**Maximum oxygen saturation**	**98.9 (97.8 - 99.9)**	**98.8 (97.4 - 99.9)**	**-0.326 (-0.542 - -0.110)**	**0.003**	**0.005**

Continue on the next page

Feature	controls	cases	coefficient	p-value	p-value*
**Median oxygen saturation**	**96.7 (95.2 - 98.1)**	**96.2 (94.1 - 97.8)**	**-0.495 (-0.728 - -0.261)**	**0.000**	**0.000**
**Minimum oxygen saturation**	**91.3 (88.0 - 93.8)**	**86.2 (82.7 - 90.3)**	**-0.483 (-0.649 - -0.318)**	**0.000**	**0.000**
**Instability oxygen saturation**	**7.0 (4.8 - 10.5)**	**11.0 (7.0 - 16.7)**	**0.432 (0.262 - 0.602)**	**0.000**	**0.000**
Use of erythrocytes transfusion	70 (0.3%)	1 (1.5%)	0.091 (-0.014 - 0.195)	0.091	0.120
Use of plasma	5 (0.0%)	0 (0.0%)	-0.219 (-359.131 - 358.693)	0.999	0.999
Use of thrombocytes	4 (0.0%)	0 (0.0%)	-0.196 (-359.115 - 358.723)	0.999	0.999
**Infusion of hydroxyethyl starch**	**112 (0.4%)**	**3 (4.5%)**	**0.159 (0.081 - 0.238)**	**0.000**	**0.000**
Contact with anesthesiologist	1311 (5.2%)	6 (9.2%)	0.139 (-0.048 - 0.325)	0.145	0.182
Overall					
**Total perioperative time**	**160.0 (126.0 - 208.0)**	**183.5 (126.0 - 243.0)**	**0.224 (0.032 - 0.416)**	**0.022**	**0.034**
**Arterial line**	**760 (3.0%)**	**12 (18.2%)**	**0.339 (0.231 - 0.447)**	**0.000**	**0.000**
**Maximum diastolic blood pressure**	**90.0 (81.0 - 100.0)**	**84.0 (71.0 - 97.0)**	**0.216 (0.086 - 0.346)**	**0.001**	**0.002**
**Minimum diastolic blood pressure**	**43.0 (37.0 - 50.0)**	**34.0 (28.0 - 40.0)**	**-0.877 (-1.087 - -0.667)**	**0.000**	**0.000**
**Instability diastolic blood pressure**	**46.0 (36.0 - 58.0)**	**48.0 (35.0 - 64.0)**	**0.299 (0.199 - 0.399)**	**0.000**	**0.000**
**Maximum mean blood pressure**	**119.0 (107.0 - 132.0)**	**116.0 (103.0 - 135.0)**	**0.241 (0.092 - 0.390)**	**0.002**	**0.003**
**Minimum mean blood pressure**	**60.0 (53.0 - 69.0)**	**48.0 (38.0 - 56.0)**	**-0.813 (-0.984 - -0.641)**	**0.000**	**0.000**
**Instability mean blood pressure**	**57.0 (43.0 - 72.0)**	**63.0 (51.0 - 84.0)**	**0.370 (0.265 - 0.476)**	**0.000**	**0.000**
**Maximum systolic blood pressure**	**163.0 (145.0 - 184.0)**	**165.0 (142.0 - 196.0)**	**0.237 (0.022 - 0.453)**	**0.031**	**0.044**
**Minimum systolic blood pressure**	**81.0 (70.0 - 93.0)**	**62.0 (54.0 - 75.0)**	**-0.837 (-1.013 - -0.660)**	**0.000**	**0.000**
**Instability systolic blood pressure**	**81.0 (58.0 - 104.0)**	**97.0 (75.0 - 135.0)**	**0.524 (0.376 - 0.673)**	**0.000**	**0.000**
**Maximum heart rate**	**98.0 (86.0 - 110.0)**	**98.5 (85.2 - 113.8)**	**0.229 (0.026 - 0.432)**	**0.027**	**0.040**
**Median heart rate**	**69.0 (62.0 - 78.0)**	**78.5 (70.0 - 87.8)**	**0.617 (0.427 - 0.807)**	**0.000**	**0.000**
**Minimum heart rate**	**53.0 (47.0 - 60.0)**	**58.0 (50.5 - 69.8)**	**0.480 (0.279 - 0.680)**	**0.000**	**0.000**
Instability heart rate	43.0 (32.0 - 55.0)	38.0 (25.0 - 54.8)	-0.042 (-0.289 - 0.205)	0.739	0.779
**Maximum oxygen saturation**	**100.0 (99.3 - 100.0)**	**100.0 (99.3 - 100.0)**	**-0.244 (-0.409 - -0.079)**	**0.004**	**0.006**
**Median oxygen saturation**	**97.3 (96.2 - 98.2)**	**97.0 (95.5 - 98.2)**	**-0.482 (-0.660 - -0.305)**	**0.000**	**0.000**
**Minimum oxygen saturation**	**89.8 (85.6 - 92.8)**	**84.9 (78.9 - 88.9)**	**-0.518 (-0.687 - -0.349)**	**0.000**	**0.000**
**Instability oxygen saturation**	**9.7 (6.7 - 14.0)**	**14.4 (10.3 - 21.1)**	**0.496 (0.324 - 0.667)**	**0.000**	**0.000**

Table 7: Summary statistic for the combination of unplanned ICU admissions and in-hospital mortality

Feature	controls	cases	coefficient	p-value	p-value*
Total number of patients	25250	229			
Preoperative					
**Age**	**55.0 (42.0 - 69.0)**	**69.0 (56.0 - 77.0)**	**0.678 (0.530 - 0.826)**	**0.000**	**0.000**
**ASA score**	**2.0 (2.0 - 2.0)**	**2.0 (2.0 - 3.0)**	**0.759 (0.620 - 0.899)**	**0.000**	**0.000**
BMI	27.1 (23.9 - 33.1)	26.0 (23.1 - 32.1)	-0.129 (-0.282 - 0.024)	0.098	0.115
Length of patient	170.0 (164.0 - 178.0)	170.0 (164.0 - 180.0)	0.029 (-0.112 - 0.171)	0.684	0.713
Weight of the patient	80.0 (69.0 - 98.0)	80.0 (65.1 - 97.0)	-0.097 (-0.246 - 0.052)	0.203	0.221
**Sex**	**9974 (39.5%)**	**124 (54.1%)**	**0.290 (0.162 - 0.418)**	**0.000**	**0.000**
**Diastolic blood pressure**	**80.0 (70.0 - 85.0)**	**75.0 (69.0 - 85.0)**	**-0.220 (-0.358 - -0.082)**	**0.002**	**0.002**
Systolic blood pressure	130.0 (120.0 - 145.0)	133.0 (120.0 - 145.0)	-0.032 (-0.176 - 0.112)	0.665	0.700
History of alcohol use	1194 (5.4%)	11 (6.6%)	0.050 (-0.089 - 0.189)	0.479	0.510
History of cerebrovascular accident	888 (4.0%)	10 (6.0%)	0.085 (-0.042 - 0.211)	0.189	0.208
History of COPD	2747 (12.4%)	22 (13.3%)	0.026 (-0.122 - 0.174)	0.732	0.751
**History of diabetes**	**2464 (11.1%)**	**37 (22.3%)**	**0.262 (0.146 - 0.378)**	**0.000**	**0.000**
**History of heart failure**	**3197 (14.4%)**	**39 (22.5%)**	**0.194 (0.068 - 0.320)**	**0.003**	**0.003**
History of heart rhythm problems	2831 (12.8%)	28 (16.9%)	0.110 (-0.027 - 0.246)	0.115	0.134
**History of hypertension**	**5944 (26.8%)**	**66 (39.8%)**	**0.262 (0.123 - 0.400)**	**0.000**	**0.000**
History of kidney failure	845 (3.8%)	10 (6.0%)	0.093 (-0.031 - 0.216)	0.141	0.159
History of smoking	4781 (21.6%)	42 (25.3%)	0.085 (-0.060 - 0.230)	0.250	0.270
**History of thrombosis**	**986 (4.5%)**	**14 (8.5%)**	**0.142 (0.028 - 0.256)**	**0.014**	**0.018**
Surgery					
**Duration in OR**	**77.0 (54.0 - 113.0)**	**121.0 (81.0 - 168.0)**	**0.362 (0.286 - 0.437)**	**0.000**	**0.000**
**Surgery duration**	**52.0 (32.0 - 80.0)**	**87.0 (52.0 - 119.0)**	**0.298 (0.223 - 0.373)**	**0.000**	**0.000**
**Elective surgery**	**2846 (11.3%)**	**60 (26.2%)**	**0.327 (0.232 - 0.421)**	**0.000**	**0.000**
**Surgery group (General)**	**12957 (51.3%)**	**169 (73.8%)**	**0.491 (0.344 - 0.639)**	**0.000**	**0.000**
**Surgery group (Gynecology)**	**2759 (10.9%)**	**6 (2.6%)**	**-0.472 (-0.724 - -0.219)**	**0.000**	**0.000**
Surgery group (Orthopedic)	3778 (15.0%)	27 (11.8%)	-0.098 (-0.242 - 0.046)	0.181	0.202
**Surgery group (Other)**	**2987 (11.8%)**	**13 (5.7%)**	**-0.258 (-0.439 - -0.078)**	**0.005**	**0.007**
**Surgery group (Urology)**	**2769 (11.0%)**	**14 (6.1%)**	**-0.199 (-0.368 - -0.030)**	**0.021**	**0.026**
**Arterial line**	**716 (2.8%)**	**36 (15.7%)**	**0.314 (0.252 - 0.375)**	**0.000**	**0.000**
**Maximum diastolic blood pressure**	**87.0 (78.0 - 97.0)**	**89.0 (78.8 - 101.0)**	**0.223 (0.152 - 0.293)**	**0.000**	**0.000**
**Minimum diastolic blood pressure**	**43.0 (37.0 - 51.0)**	**36.5 (31.0 - 44.0)**	**-0.652 (-0.788 - -0.515)**	**0.000**	**0.000**
**Instability diastolic blood pressure**	**43.0 (32.0 - 54.0)**	**49.0 (40.8 - 65.0)**	**0.285 (0.226 - 0.343)**	**0.000**	**0.000**
**Maximum mean blood pressure**	**114.0 (102.0 - 128.0)**	**117.0 (104.0 - 132.0)**	**0.245 (0.163 - 0.328)**	**0.000**	**0.000**
**Minimum mean blood pressure**	**61.0 (53.0 - 70.0)**	**52.0 (45.0 - 59.2)**	**-0.645 (-0.760 - -0.529)**	**0.000**	**0.000**
**Instability mean blood pressure**	**52.0 (38.0 - 68.0)**	**62.5 (50.0 - 81.0)**	**0.347 (0.281 - 0.412)**	**0.000**	**0.000**
**Maximum systolic blood pressure**	**157.0 (138.0 - 177.0)**	**159.0 (140.0 - 183.2)**	**0.227 (0.110 - 0.345)**	**0.000**	**0.000**
**Minimum systolic blood pressure**	**82.0 (71.0 - 94.0)**	**70.0 (59.8 - 81.0)**	**-0.673 (-0.792 - -0.553)**	**0.000**	**0.000**
**Instability systolic blood pressure**	**73.0 (50.0 - 97.0)**	**87.5 (69.0 - 116.2)**	**0.457 (0.364 - 0.551)**	**0.000**	**0.000**
**Maximum heart rate**	**95.0 (83.0 - 107.0)**	**100.0 (88.0 - 112.0)**	**0.198 (0.083 - 0.313)**	**0.001**	**0.001**
**Median heart rate**	**66.0 (59.0 - 75.0)**	**71.0 (63.0 - 81.0)**	**0.390 (0.278 - 0.502)**	**0.000**	**0.000**
**Minimum heart rate**	**54.0 (48.0 - 61.0)**	**56.0 (49.0 - 64.0)**	**0.199 (0.077 - 0.322)**	**0.001**	**0.002**
Instability heart rate	40.0 (29.0 - 52.0)	41.0 (31.0 - 52.0)	0.096 (-0.027 - 0.219)	0.125	0.144
Maximum oxygen saturation	99.9 (99.1 - 100.0)	99.8 (99.1 - 100.0)	-0.101 (-0.216 - 0.014)	0.086	0.102
**Median oxygen saturation**	**97.7 (96.7 - 98.4)**	**97.6 (96.5 - 98.5)**	**-0.188 (-0.303 - -0.073)**	**0.001**	**0.002**
**Minimum oxygen saturation**	**93.4 (89.4 - 95.4)**	**92.5 (87.7 - 94.9)**	**-0.190 (-0.299 - -0.082)**	**0.001**	**0.001**
**Instability oxygen saturation**	**5.9 (4.0 - 9.9)**	**6.8 (4.5 - 11.3)**	**0.179 (0.070 - 0.288)**	**0.001**	**0.002**
**Narcosis type (General)**	**19394 (76.8%)**	**159 (69.4%)**	**-0.159 (-0.279 - -0.040)**	**0.009**	**0.011**
**Narcosis type (General + Epidural)**	**1076 (4.3%)**	**57 (24.9%)**	**0.414 (0.351 - 0.477)**	**0.000**	**0.000**
Narcosis type (Other)	1054 (4.2%)	4 (1.7%)	-0.179 (-0.376 - 0.019)	0.076	0.092
**Narcosis type (Spinal)**	**3726 (14.8%)**	**9 (3.9%)**	**-0.510 (-0.746 - -0.274)**	**0.000**	**0.000**
**Use of erythrocytes transfusion**	**124 (0.5%)**	**13 (5.7%)**	**0.183 (0.140 - 0.226)**	**0.000**	**0.000**
**Use of plasma**	**13 (0.1%)**	**2 (0.9%)**	**0.069 (0.033 - 0.105)**	**0.000**	**0.000**
**Use of ephedrine**	**11929 (47.2%)**	**135 (59.0%)**	**0.236 (0.104 - 0.368)**	**0.000**	**0.001**
**Use of phenylephrine**	**4485 (17.8%)**	**123 (53.7%)**	**0.647 (0.546 - 0.748)**	**0.000**	**0.000**
**Use of vasopressor pump**	**1183 (4.7%)**	**32 (14.0%)**	**0.255 (0.174 - 0.335)**	**0.000**	**0.000**
**Use of thrombocytes**	**6 (0.0%)**	**2 (0.9%)**	**0.064 (0.036 - 0.092)**	**0.000**	**0.000**
**Infusion of hydroxyethyl starch**	**634 (2.5%)**	**25 (10.9%)**	**0.248 (0.181 - 0.315)**	**0.000**	**0.000**
Recovery					

Continue on the next page

Feature	controls	cases	coefficient	p-value	p-value*
**Duration in PACU**	**74.0 (59.0 - 96.0)**	**98.5 (74.0 - 139.0)**	**0.463 (0.385 - 0.540)**	**0.000**	**0.000**
**Arterial line**	**476 (1.9%)**	**27 (11.8%)**	**0.270 (0.213 - 0.327)**	**0.000**	**0.000**
**Maximum diastolic blood pressure**	**81.0 (72.0 - 90.0)**	**81.0 (70.0 - 94.0)**	**0.140 (0.048 - 0.232)**	**0.003**	**0.004**
**Minimum diastolic blood pressure**	**63.0 (54.0 - 72.0)**	**59.0 (47.0 - 69.0)**	**-0.360 (-0.485 - -0.236)**	**0.000**	**0.000**
**Instability blood pressure**	**16.0 (11.0 - 23.0)**	**20.5 (14.0 - 29.2)**	**0.194 (0.138 - 0.250)**	**0.000**	**0.000**
**Maximum mean blood pressure**	**107.0 (96.0 - 119.0)**	**109.5 (97.0 - 123.2)**	**0.175 (0.072 - 0.279)**	**0.001**	**0.001**
**Minimum mean blood pressure**	**87.0 (77.0 - 98.0)**	**80.5 (69.0 - 95.0)**	**-0.359 (-0.474 - -0.244)**	**0.000**	**0.000**
**Instability mean blood pressure**	**18.0 (13.0 - 27.0)**	**25.0 (17.0 - 40.0)**	**0.234 (0.178 - 0.290)**	**0.000**	**0.000**
**Maximum systolic blood pressure**	**148.0 (131.0 - 166.0)**	**154.0 (136.0 - 176.5)**	**0.216 (0.093 - 0.339)**	**0.001**	**0.001**
**Minimum systolic blood pressure**	**120.0 (105.0 - 136.0)**	**112.5 (92.8 - 131.0)**	**-0.370 (-0.491 - -0.249)**	**0.000**	**0.000**
**Instability systolic blood pressure**	**25.0 (17.0 - 37.0)**	**37.0 (23.0 - 58.0)**	**0.322 (0.259 - 0.385)**	**0.000**	**0.000**
**Maximum heart rate**	**88.0 (77.0 - 100.0)**	**94.5 (83.0 - 105.0)**	**0.311 (0.197 - 0.425)**	**0.000**	**0.000**
**Median heart rate**	**71.5 (63.0 - 81.5)**	**80.0 (71.0 - 89.0)**	**0.464 (0.345 - 0.584)**	**0.000**	**0.000**
**Minimum heart rate**	**63.0 (55.0 - 73.0)**	**70.5 (62.0 - 80.0)**	**0.447 (0.325 - 0.568)**	**0.000**	**0.000**
Instability heart rate	22.0 (15.0 - 31.0)	21.0 (14.2 - 30.0)	-0.024 (-0.162 - 0.115)	0.736	0.751
**Maximum oxygen saturation**	**98.9 (97.8 - 99.9)**	**98.8 (97.6 - 99.9)**	**-0.184 (-0.307 - -0.061)**	**0.003**	**0.004**
**Median oxygen saturation**	**96.7 (95.2 - 98.1)**	**96.4 (94.6 - 97.8)**	**-0.257 (-0.386 - -0.128)**	**0.000**	**0.000**
**Minimum oxygen saturation**	**91.3 (88.1 - 93.8)**	**89.1 (84.7 - 92.0)**	**-0.325 (-0.426 - -0.225)**	**0.000**	**0.000**
**Instability oxygen saturation**	**7.0 (4.8 - 10.5)**	**8.8 (6.4 - 13.5)**	**0.291 (0.188 - 0.393)**	**0.000**	**0.000**
**Use of erythrocytes transfusion**	**67 (0.3%)**	**4 (1.7%)**	**0.100 (0.047 - 0.154)**	**0.000**	**0.000**
Use of plasma	5 (0.0%)	0 (0.0%)	-0.236 (-359.148 - 358.676)	0.999	0.999
Use of thrombocytes	4 (0.0%)	0 (0.0%)	-0.211 (-359.130 - 358.708)	0.999	0.999
**Infusion of hydroxyethyl starch**	**108 (0.4%)**	**7 (3.1%)**	**0.134 (0.082 - 0.186)**	**0.000**	**0.000**
**Contact with anesthesiologist**	**1290 (5.1%)**	**27 (12.1%)**	**0.207 (0.117 - 0.297)**	**0.000**	**0.000**
Overall					
**Total perioperative time**	**160.0 (126.0 - 207.0)**	**218.0 (157.0 - 306.0)**	**0.467 (0.387 - 0.547)**	**0.000**	**0.000**
**Arterial line**	**736 (2.9%)**	**36 (15.7%)**	**0.313 (0.251 - 0.375)**	**0.000**	**0.000**
**Maximum diastolic blood pressure**	**90.0 (81.0 - 100.0)**	**92.0 (82.0 - 105.2)**	**0.229 (0.160 - 0.298)**	**0.000**	**0.000**
**Minimum diastolic blood pressure**	**43.0 (37.0 - 50.0)**	**36.0 (30.0 - 44.0)**	**-0.602 (-0.732 - -0.472)**	**0.000**	**0.000**
**Instability diastolic blood pressure**	**46.0 (36.0 - 58.0)**	**52.5 (42.0 - 70.0)**	**0.281 (0.221 - 0.341)**	**0.000**	**0.000**
**Maximum mean blood pressure**	**119.0 (107.0 - 131.0)**	**121.5 (109.0 - 137.2)**	**0.258 (0.179 - 0.336)**	**0.000**	**0.000**
**Minimum mean blood pressure**	**61.0 (53.0 - 69.0)**	**52.0 (44.0 - 59.2)**	**-0.599 (-0.708 - -0.491)**	**0.000**	**0.000**
**Instability mean blood pressure**	**57.0 (43.0 - 72.0)**	**67.0 (54.0 - 87.2)**	**0.341 (0.276 - 0.405)**	**0.000**	**0.000**
**Maximum systolic blood pressure**	**163.0 (145.0 - 184.0)**	**170.0 (149.8 - 196.0)**	**0.272 (0.158 - 0.385)**	**0.000**	**0.000**
**Minimum systolic blood pressure**	**81.0 (71.0 - 93.0)**	**69.5 (59.0 - 81.0)**	**-0.622 (-0.733 - -0.512)**	**0.000**	**0.000**
**Instability systolic blood pressure**	**81.0 (58.0 - 104.0)**	**98.0 (76.0 - 128.0)**	**0.458 (0.369 - 0.547)**	**0.000**	**0.000**
**Maximum heart rate**	**97.0 (86.0 - 110.0)**	**103.0 (90.0 - 114.0)**	**0.218 (0.106 - 0.330)**	**0.000**	**0.000**
**Median heart rate**	**69.0 (62.0 - 78.0)**	**76.0 (68.0 - 85.0)**	**0.450 (0.337 - 0.562)**	**0.000**	**0.000**
**Minimum heart rate**	**53.0 (47.0 - 60.0)**	**55.0 (49.0 - 64.0)**	**0.262 (0.143 - 0.382)**	**0.000**	**0.000**
Instability heart rate	43.0 (32.0 - 55.0)	44.0 (33.0 - 57.0)	0.095 (-0.027 - 0.217)	0.129	0.147
**Maximum oxygen saturation**	**100.0 (99.3 - 100.0)**	**100.0 (99.2 - 100.0)**	**-0.134 (-0.243 - -0.026)**	**0.015**	**0.019**
**Median oxygen saturation**	**97.3 (96.3 - 98.2)**	**97.1 (95.8 - 98.1)**	**-0.258 (-0.374 - -0.143)**	**0.000**	**0.000**
**Minimum oxygen saturation**	**89.8 (85.6 - 92.8)**	**87.5 (83.3 - 91.0)**	**-0.283 (-0.390 - -0.177)**	**0.000**	**0.000**
**Instability oxygen saturation**	**9.7 (6.7 - 14.0)**	**11.6 (8.4 - 16.2)**	**0.270 (0.163 - 0.377)**	**0.000**	**0.000**

B Hyperparameters

Table 8: Grid search parameters

Parameter	Grid values
Logistic Regression	
C	0.001, 0.01, 0.03, 0.07, 0.13, 0.25, 0.5, 1
Support Vector Machine	
C	0.001, 0.01, 0.03, 0.07, 0.13, 0.25, 0.5, 1
γ	0.0001, 0.001, 0.01, 0.1, 1
Random Forest	
Maximum depth	2, 4, 8, 16, 32, None
Minimum samples before split	2, 4, 8, 16, 32, 64
Minimum samples per leaf	1, 2, 4, 8, 16, 32
Extreme Gradient Boosting	
Maximum depth	2, 4, 6, 8, 10
Minimum child weight	0.01, 0.1, 1, 10, 100
γ	0.01, 0.1, 1, 10, 100
Learning rate	0.1, 0.2, 0.3, 0.4

C Resampling technique

Table 9: Multi-variate analysis of unanticipated ICU admissions and in-hospital mortality using resampling

Method	AUROC	AUPRC	AUKC	Precision	Sensitivity	Kappa	F1 score
End-point: Unanticipated ICU admissions							
LR	82.9% (2.7%)	5.8% (2.5%)	2.1% (0.3%)	9.8% (4.1%)	13.2% (5.6%)	0.10 (0.05)	11.2% (4.6%)
End-point: In-hospital mortality							
LR	90.0% (5.7%)	5.7% (3.1%)	1.6% (0.5%)	6.7% (2.9%)	26.1% (11.0%)	0.10 (0.04)	10.6% (4.4%)
End-point: Unanticipated ICU admissions & in-hospital mortality							
LR	82.8% (3.1%)	6.8% (2.8%)	2.5% (0.3%)	10.2% (3.7%)	11.5% (4.6%)	0.10 (0.04)	10.7% (4.0%)

Area Under the Receiver Operating Characteristic curve (AUROC), Area Under the Precision-Recall curve (AUPRC), Area Under the Kappa curve (AUKC).

D SHAP values

Table 10: Feature importance of multi-variate analysis

Feature	Phase	Importance
Unanticipated ICU Admissions		
Surgery group (General)	Surgery	0.307 (0.116)
Use of phenylephrine	Surgery	0.175 (0.154)
Minimum heart rate	Recovery	0.135 (0.123)
Sex	Preoperative	0.063 (0.063)
In-Hospital Mortality		
Age	Preoperative	0.375 (0.275)
History of Hypertension	Preoperative	0.172 (0.167)
Median heart rate	Surgery	0.152 (0.148)
Use of phenylephrine	Surgery	0.139 (0.137)
Sex	Preoperative	0.098 (0.091)
Combination		
Surgery group (General)	Surgery	0.293 (0.066)
Age	Preoperative	0.237 (0.169)
Use of Fenyl	Surgery	0.178 (0.155)
Sex	Preoperative	0.117 (0.061)
Median heart rate	Surgery	0.088 (0.082)
